# Flexible artificial lateral line based on luminous flux for underwater velocity vector estimation

**DOI:** 10.1093/nsr/nwag337

**Published:** 2026-06-05

**Authors:** Xintao Wang, Zhengwei Li, Zhuoliang Zhang, Junfeng Fan, Yaming Ou, Xiangyu Sun, Min Tan, Long Cheng, Chao Zhou

**Affiliations:** Key Laboratory of Cognition and Decision Intelligence for Complex Systems, Institute of Automation, Chinese Academy of Sciences, Beijing 100190, China; School of Artificial Intelligence, University of Chinese Academy of Sciences, Beijing 100049, China; School of Artificial Intelligence, University of Chinese Academy of Sciences, Beijing 100049, China; Key Laboratory of Cognition and Decision Intelligence for Complex Systems, Institute of Automation, Chinese Academy of Sciences, Beijing 100190, China; Key Laboratory of Cognition and Decision Intelligence for Complex Systems, Institute of Automation, Chinese Academy of Sciences, Beijing 100190, China; Key Laboratory of Cognition and Decision Intelligence for Complex Systems, Institute of Automation, Chinese Academy of Sciences, Beijing 100190, China; School of Artificial Intelligence, University of Chinese Academy of Sciences, Beijing 100049, China; Key Laboratory of Cognition and Decision Intelligence for Complex Systems, Institute of Automation, Chinese Academy of Sciences, Beijing 100190, China; School of Artificial Intelligence, University of Chinese Academy of Sciences, Beijing 100049, China; Key Laboratory of Cognition and Decision Intelligence for Complex Systems, Institute of Automation, Chinese Academy of Sciences, Beijing 100190, China; School of Artificial Intelligence, University of Chinese Academy of Sciences, Beijing 100049, China; Key Laboratory of Cognition and Decision Intelligence for Complex Systems, Institute of Automation, Chinese Academy of Sciences, Beijing 100190, China

**Keywords:** artificial lateral line, flexible sensor, luminous flux sensor, flow velocity sensing, trajectory estimation

## Abstract

When fish swim, a specific ‘water flow field’ forms around their bodies. The lateral line system can provide real-time feedback on flow field variations through the stimulation of hair cells by water currents. Therefore, this paper developed a flexible artificial lateral line (ALL) sensor unit based on the principle of luminous flux that measures flow velocity vector. The sensor employs a dual-layer inverted cup-shaped rocker design. Water flow impacts the rocker, compressing the flexible silicone spring and converting flow velocity changes into variations in luminous flux received by photosensitive units in multiple directions, thereby achieving local flow velocity vector sensing. To address traditional modeling challenges posed by large deformations, nonlinear mechanics, and coupling characteristics in flexible materials, a deep neural network-based flow velocity perception algorithm named CLANN is proposed. This algorithm not only facilitates calibration of flow velocity vectors but also enables multi-sensor data fusion for more accurate flow velocity prediction. Finally, the proposed ALL sensor unit was integrated onto an underwater robotic platform. Under attitude disturbance conditions, the sensor was fused with an inertial measurement unit to achieve multi-sensor fusion estimation of the robot’s velocity vector. Results indicate that the measured velocity vector exhibits a mean absolute error of 0.048 m/s in magnitude and 16.49° in direction, with a linearity coefficient ($R^2$) of 0.896. Furthermore, the robot can estimate its own trajectory under different motion states with an error of 0.284 m.

## Teaser

Flexible luminous flux-based artificial lateral line sensor enables underwater velocity vector estimation under attitude disturbance conditions via neural network algorithms.

## INTRODUCTION

Underwater robotics technology has evolved alongside deepening exploration of the oceans, playing a crucial role in marine resource exploration [1], environmental monitoring [2], and ecological conservation [3,4]. Acquiring flow field information is one of the key capabilities for underwater robots to perform tasks in complex marine environments [5], but the complex marine environment also poses significant challenges to underwater perception [6,7].

Inspired by bionics, bionic sensors offer novel approaches for capturing flow field information [8–11]. Aquatic organisms achieve effective navigation and obstacle avoidance in complex aquatic environments through precise environmental perception [12,13]. Many fish utilize an organ known as the lateral line system to detect hydrodynamic changes such as pressure and flow velocity. The lateral line system comprises superficial neuromasts and canal neuromasts (CN) [14,15], exhibiting high sensitivity to environmental changes. This sensory system plays a crucial role in helping fish evade predators and avoid capture in low-visibility marine environments [16,17]. Based on this, researchers have developed artificial lateral line (ALL) sensors with different sensing mechanisms, which can detect subtle water flow changes and effectively acquire environmental information in complex environments [18,19].

Flow velocity sensing forms the foundation for underwater robots to perform trajectory estimation, obstacle detection, and motion planning [20]. It also serves as the core support enabling robots to adapt to complex marine environments and enhance autonomous operation capabilities [3]. Accurate flow velocity sensing not only provides robots with real-time flow field information but also enables them to anticipate flow changes and optimize path planning strategies [21]. However, traditional acoustic devices suffer from large size, complex installation, and susceptibility to seabed irregularities [22]. Vision technologies, primarily relying on visual perception, offer high sensitivity but suffer from reduced measurement range due to water turbidity and limited robustness stemming from light scattering in aquatic media [23,24]. To address these challenges, this paper explores an ALL sensor. By mimicking the sensory architecture of the superficial neuromasts, it enables measurement of complex flow fields.

Optical sensors offer advantages such as high sensitivity, high precision, strong resistance to electromagnetic interference, and rapid response times [25,26]. They are also being integrated with bionic lateral lines. Optical ALL encompasses vision-based ALL and fiber-optic ALL. As early as 2011, K Adrian *et al.* [27] developed a fiber-optic ALL channel mimicking the CN. A silicon rod in the middle of the canal tilts under water flow impact to change the optical path, thereby capturing variations and detecting vibration sources. Wiesmayr et al. [28] further designed a flow sensor simulating lateral line hair cells based on the CN. By connecting a light-emitting diode (LED) and photodiode via a polydimethylsiloxane (PDMS) waveguide, it senses changes in luminous flux through waveguide bending caused by water flow impact, enabling flow detection. Li *et al.* [29] proposed a 3D nano-printed biomimetic fiber optic neurone that enables integrated, highly sensitive underwater multi-physics detection through opto-mechanical synergy, achieving an acoustic sensitivity of 172.24 V/kPa, an ocean turbulence sensitivity of 8560.72 nm/(m/s), and a wide-angle response of 0–180°.

The ALL sensors include rigid sensors and flexible sensors. These rigid sensors are characterized by high material hardness and strong mechanical stability [30]. They exhibit excellent resistance to mechanical impact and wear, making them suitable for long-term complex underwater operating conditions [31]. However, constrained by the high elastic modulus and fixed structural characteristics of rigid materials, their deformation threshold is relatively high [32]. This makes it difficult for them to produce a significant response to weak underwater flow fields (such as low flow velocities or low-amplitude vortices), resulting in insufficient detection accuracy for low-speed flow fields or minute disturbances [18]. In contrast, flexible sensors leverage low elastic modulus and micro-deformation adaptability to precisely capture minute mechanical disturbances induced by weak flow fields [33]. Furthermore, the high surface compliance of flexible materials allows for superior adhesion to the hull surfaces of underwater robotic vehicles [34]. This reduces flow separation phenomena and lowers the drag coefficient during navigation, driving the expanding application of flexible sensors in lateral line sensor technology.

Another limitation of ALL sensors lies in the fact that most existing ALL sensor units can only detect the magnitude of flow velocity [10,27,28], while the perception of flow velocity vectors largely relies on signal fusion and estimation from array-based sensors [18,32,35]. Research on directly measuring flow velocity vectors using a single sensor unit is scarce [9,36], and the existing studies still suffer from several limitations. Particularly for ALL sensors employing cantilever beam structures, their structural characteristics make it challenging for underwater robots equipped with ALL sensors to derive their velocity vectors. This limitation prevents robots from performing complex tasks such as autonomous navigation and underwater path planning.

In response to the limitations of the aforementioned ALL sensors, inspired by the superficial neuromast, this paper proposes a flexible ALL sensor based on luminous flux. This work contributes in the following three aspects. A flexible bionic ALL sensor based on luminous flux was proposed that enables vector velocity measurement. The sensor addresses the issue that most ALLs rely on array-based velocity vector calculation, while also overcoming the limitations of existing single-unit vector measurement schemes to a certain extent. By integrating flexible sensing capability and leveraging the differentiated deformation mechanism within the sensor unit’s internal structure, a single sensor unit can achieve vector velocity sensing while meeting the goals of compact size and lightweight design. A hybrid neural network algorithm based on cross-loss functions named CLANN was proposed. This algorithm mitigates the information loss issue in traditional recurrent neural networks when handling ultra-long time series and partially resolves the coupling problem of high-dimensional heterogeneous data. It can integrate data from the ALL sensor and inertial measurement unit (IMU) to achieve accurate estimation of the global velocity vector under changes in the body coordinate system. The designed ALL sensor was integrated into a Remotely Operated Vehicle (ROV) platform to conduct multi-motion experiments under the condition of robotic attitude disturbances. Considering attitude disturbances, the experiments addressed two key challenges: velocity prediction and multi-sensor fusion. Three typical two-dimensional motion scenarios—linear motion, circular motion, and irregular motion—were designed to validate the feasibility and effectiveness of the proposed sensor and neural network algorithm.

## RESULTS AND DISCUSSION

### Principle of the ALL sensor

**Figure 1.**
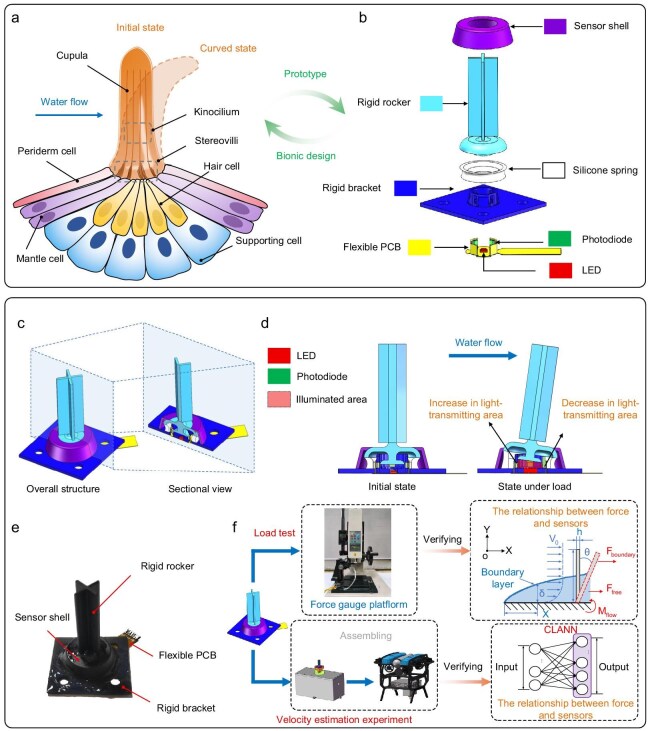
Schematic diagram of the lateral line sensor. (a) Bionic inspiration: superficial neuromast. (b) Structure of the ALL sensor. (c) Overall view and cross-section of the ALL sensor. (d) Schematic Diagram of the ALL sensor’s principle. The diagram illustrates the state changes of the sensor before and after force application. The selected cross-section corresponds to the sectional view in Fig. 1c. (e) Physical diagram of the sensor. The diagram shows some modules of the sensor, with a silicone spring inside the sensor. (f) Comprehensive overview of the ALL sensor research, including load testing and velocity estimation experiments using the proposed CLANN algorithm.

Figure 1d illustrates the basic principle of the sensor. The flexible shell blocks external light sources, ensuring that the luminous flux received by the photosensitive units originates entirely from the internal light source. The rigid rocker itself is non-transparent due to a light-blocking coating. In the initial unloaded state, the inner layer of the rocker partially obstructs some parts of photosensitive units, establishing an initial value for each photosensitive unit. The four photosensitive units are uniformly distributed around a 360° circumference, with adjacent units spaced at 90°. Two photosensitive units aligned in the same direction form one group, creating two mutually perpendicular sets. Taking the orientation of one photosensitive unit as an example: when water flows strike the rigid rocker from the side, the fluid force acts upon the rigid rocker and is transmitted through its outer layer to the flexible silicone spring beneath, causing deformation. The silicone spring on the upstream side stretches while the opposite side within the same group compresses, tilting the entire rigid rocker. Under force, the upstream inner layer of the rocker moves upward, increasing the luminous flux received by the photosensitive unit and correspondingly raising its voltage value. Conversely, the opposite inner layer of the rocker further obstructs light propagation, reducing the luminous flux and decreasing its voltage value. As flow velocity and direction change, the hydraulic forces acting on the rocker vary, causing the four photosensitive units to exhibit distinct response values. By calibrating the relationship between voltage values and flow velocity using appropriate methods, the sensor achieves its flow velocity sensing capability.

### Structural design of the ALL sensor

As shown in Fig. 1b, the designed ALL sensor unit consists of five modules: (i) flexible shell, (ii) silicone spring, (iii) opaque rigid rocker, (iv) bracket, and (v) flexible printed circuit board (FPCB). The sensor base is a three-dimensional bracket with an internal cavity and grooves on the bottom and four sides. The bracket extends to support a mounting plate, whose dimensions can be adjusted for different application scenarios to secure the sensor onto various robotic surfaces. The FPCB comprises a core section and an extension section. The core section houses four photosensitive units (photodiodes) and one LED, while the extension section solely serves to lengthen the pins. After bonding to the bracket, the LED resides within the bracket’s bottom groove, while the four photosensitive units are positioned within the side grooves. The LED brightness can be adjusted via an external variable resistor to ensure appropriate initial voltage values for each unit, preventing ‘dead zones’. The silicone spring adopts a ring shape with a concave surface structure on the outer ring, enabling more sensitive deformation. The rigid rocker comprises upper and lower sections. The upper cross-shaped structure detects water flow impact, while the lower section features an inverted double-layer cup structure with inner and outer layers. The silicone spring encircles the outer perimeter of the three-dimensional bracket, firmly bonded to both the bracket and the outer layer of the upper contact. After bonding, the inner layer of the rocker remains suspended within the cavity, partially obscuring the photosensitive units. The flexible shell adopts an inverted cup-shaped structure with a hollow top. It is fitted over the rigid contact to provide waterproofing and light shielding. Figure 1c displays the assembled sensor and its cross-sectional view. Specific sensor parameters are listed in Table S1. Due to the irregular structures of individual sensor modules, the table indicates the maximum dimensions for the entire sensor and each module, excluding extended sections of the FPCB and bracket.

### Velocity estimation method based on CLANN

#### Model framework

Traditional machine learning methods possess certain advantages in sensor modeling, but when measuring velocity vectors, they face challenges associated with multiple inputs and multiple outputs [37]. Compared to deep learning, traditional machine learning exhibits certain limitations. Specifically, for the sensors discussed in this paper, the task involves simultaneously measuring the readings from four photosensors and an IMU in real time and outputting a velocity vector. This task is fundamentally a process of feature extraction from high-dimensional heterogeneous data and decoupling coupled relationships. Traditional machine learning not only relies on manually designed features but also lacks sufficient modeling capability for the spatiotemporal coupling characteristics of sensor signals under turbulent flow conditions. In contrast, deep learning’s distinct advantages in handling high-dimensional data, complex nonlinear relationships, and automatic feature extraction make it the preferred solution for modeling ALL sensors.

**Figure 2.**
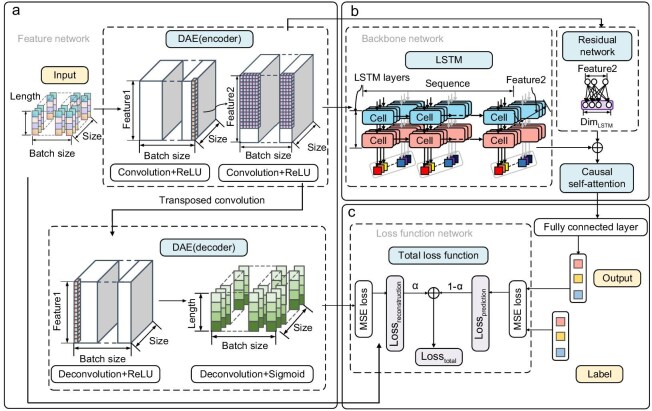
The proposed algorithm framework diagram. (a) Feature network: employs a denoising autoencoder with an embedded convolutional neural network as its core. (b) Backbone network: utilizes an LSTM as its core, fully connected to a residual network, with output processed through a causal self-attention mechanism. (c) Loss function network: incorporates prediction loss and reconstruction loss, weighted to yield the total loss.

Based on this, this paper combines the advantages of structures such as LSTM and fully connected neural networks to design a hybrid neural network model with a cross-loss function. This model achieves calibration of the proposed ALL sensor and completes velocity estimation. The model primarily consists of three modules: the feature network (FN), the backbone network (BN), and the loss function network (LFN). The model block diagram is shown in Fig. 2.

As shown in Fig. 2a, the feature network adopts a denoising autoencoder (DAE) framework, embedding a convolutional neural network within the DAE. As a variant of autoencoders, DAE aims to reconstruct the original input from corrupted data. For ALL sensor outputs contaminated with underwater noise, DAE can learn more representative features, thereby enhancing the model’s robustness against noise.

The entire DAE consists of two components: an encoder and a decoder, typically constructed using fully connected layers. The sensor data received consists of a sequence from four photosensitive units over a specific time interval. Considering that using a recurrent neural network (RNN) alone struggles to explicitly model inter-channel relationships, the model replaces the fully connected layer of the traditional DAE with a convolutional neural network (CNN), fully leveraging CNN’s spatial feature extraction capabilities. To enhance information extraction, this paper employs a ‘dimensionality expansion’ approach. Multi-channel time series are arranged in parallel according to their sampling order, forming a two-dimensional sequence. This facilitates consideration of coupling effects between different photosensitive units, aligning with the physical phenomenon where all photosensitive units exhibit corresponding changes when water flow strikes the sensor from a specific direction.

Figure 2b illustrates the basic architecture of the backbone network, which consists of multiple layers of long short-term memory (LSTM), residual network (ResNet), and a causal self-attention (CSA). LSTM was specifically designed to address the vanishing/exploding gradient issues that traditional RNNs encounter when processing long sequence data. Adding ResNet to the model helps preserve the features of the original data and mitigates the information loss issue that LSTM encounters in extremely long sequences. Furthermore, a causal self-attention is introduced on this foundation. It overcomes the limitations of LSTM’s ‘local recursion’, enhancing the model’s ability to capture complex temporal features.

The loss function network comprises prediction loss and reconstruction loss. The output of the backbone network passes through a fully connected layer to produce the final output. Comparing this output with the ground truth label yields the prediction loss. Comparing the output of the feature network with the original data yields the reconstruction loss. Both prediction loss and reconstruction loss are calculated using mean squared error (MSE). Weighting these two losses together yields the total loss. The total loss serves as the optimization objective for gradient descent:


(1) \begin{eqnarray*}
{\rm Loss}_{\rm total} &=& (1-\alpha ) \times {\rm Loss}_{\rm prediction} \\
&& +\, \alpha \times {\rm Loss}_{\rm reconstruction,}
\end{eqnarray*}

where $\alpha$ is the weighting coefficient, manually adjusted, ${\rm Loss}_{\rm prediction}$ and ${\rm Loss}_{\rm reconstruction}$ represent prediction error and reconstruction error, respectively.

Reconstruction loss quantifies a DAE’s ability to recover original data from noisy inputs; smaller errors indicate more robust features learned by the model. By minimizing the reconstruction loss, the DAE compels the hidden layer to learn key data features, with error calculation directly driving this process. Balancing the weights of reconstruction loss and prediction loss allows simultaneous consideration of the model’s predictive capability and denoising ability, further enhancing its feature extraction capacity. The structural diagram is shown in Fig. 2c. Finally, the parameters of the neural network model proposed in this paper are presented as shown in Table S2.

#### Data preparation

The size and diversity of the training dataset are key indicators for evaluating a model’s generalization ability. To validate the CLANN model’s effectiveness, a total of 1.166 h of continuous multisensor data was subsequently collected, covering the full range of operational scenarios for the underwater robot under varying flow velocities, movement modes, and environmental conditions. For further details, please refer to the Table S6.

To enhance the model generalization performance, data collection was carefully designed to cover flow velocities ranging from 0 to 0.4 m/s in the water tank environment. A small amount of abnormal working condition data is also included to improve the robustness of the proposed model.

It should be noted that the ALL sensors in this paper undergo adaptive sliding window mean filtering and normalization preprocessing before being fed into the CLANN model. specific steps are detailed in the Note S4.

### Relationship between force and sensor

This subsection serves as the initial phase of the ALL sensor velocity vector estimation study, with its core objective centered on validating the effectiveness of the sensor principle, signal response characteristics, and directional perception capabilities. Furthermore, it should be noted that to simulate the impact of water flow on the rocker, the force analysis in this section is conducted within a plane perpendicular to the rocker, disregarding forces acting along the axial direction of the rocker.

**Figure 3.**
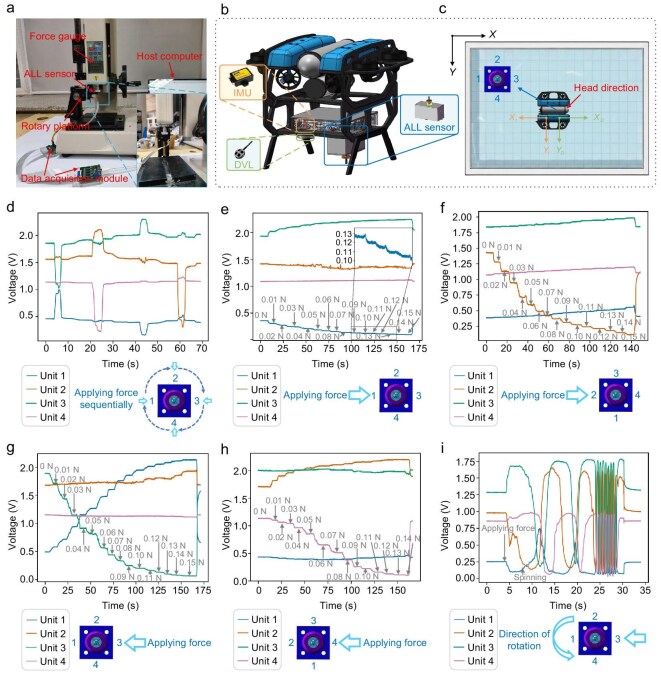
Experiment platform and relationship between force and sensor experiment results. (a) Force gauge platform. The bottom right corner shows the platform’s side view. The force gauge is vertically fixed at the center of the platform, with its lower end serving as the rocker for force application. The sensor is mounted vertically on a rotating platform, while the circular platform is fixed to the force gauge platform and allows for adjustable rotation angles. The force gauge has a measurement range of 0–2 N, a resolution of 0.0001 N, and an accuracy of $\pm 1\%$. The platform height can be adjusted by manually operating a lever, enabling vertical movement of the force gauge. (b) ROV underwater platform. Equipped with IMU, DVL, and the proposed ALL sensor. The sensor is mounted on a metal platform, and the entire assembly is secured to the bottom of the ROV, with a sampling rate of 22 Hz. Inside the platform are housed the sensor’s power supply module and data acquisition module. Data is transmitted to the ROV’s main host for frame synchronization with the IMU. The IMU is installed within the ROV’s main hull compartment, operating at a sampling rate of 100 Hz. The DVL operates at a sampling rate of approximately 5 Hz and is used solely as a velocity reference in this section. (c) Coordinate system rules for different Sensors. The upper left corner of the tank is defined as the origin. The horizontal direction to the right is the positive *X*-axis, and the vertical direction downward is the positive *Y*-axis. The coordinate systems for the DVL and IMU are defined as $OX_{\rm D}Y_{\rm D}$ and $OX_{\rm I}Y_{\rm I}$, respectively, where $X_D$ aligns with the ROV’s head direction. The underwater experimental environment consists of a water tank measuring $5\, {\rm m}\times 4\, {\rm m}\times 1.2\, {\rm m}$. (d) Sensor principle verification experiment. The bottom of each subfigure shows a top view of the sensor and a description of the applied force. The following e–i have identical representations. (e) Step force application experiment for unit 1. (f) Step force application experiment for unit 2. (g) Step force application experiment for unit 3. (h) Step force application experiment for unit 4. (i) Directional change perception experiment.

Figure 3 shows the initial voltage differences among the four photosensitive units in their stationary state. This is a normal occurrence and does not affect the sensor’s performance. It is caused by the fact that, in practice, it is difficult to achieve the high precision required by the standards during sensor manufacturing, and there are certain installation tolerances in the rocker and photosensitive units, which lead to this phenomenon.

(i) Principle verification experiment: As shown in Fig. 3d, under initial no-force conditions, the rocker partially obscures the photosensitive units, each of which has an initial value. Taking unit 1 as an example: when a lateral force perpendicular to the rocker is applied in the direction from unit 1 to unit 3, the rocker will tilt toward unit 3. This increases the luminous flux received by unit 1, raising its voltage value. Correspondingly, the luminous flux received by unit 3 on the opposite side decreases, lowering its voltage value. Applying perpendicular lateral forces to the sensor in sequence from unit 1 to unit 4 will yield the same pattern of changes in the remaining directions.

(ii) Step force experiment: Figure 3e–h show the results of step forces applied to the ALL sensor in four directions. The force direction is perpendicular to the rocker, with the center of application located at the rocker tip. The force gradually increases from 0 N to 0.15 N, pausing at intervals of 0.01 N. It can be observed that as the force increases, the voltage values in different directions of the sensor do not change abruptly but exhibit corresponding variations with the gradual increase in force. Even for minute changes from 0 N to 0.01 N, the sensor triggers a continuous response, demonstrating excellent force sensitivity. Additionally, the force–voltage response of the sensor exhibits significant nonlinear characteristics. This occurs because the sensor’s voltage variation relies on the deformation of its silicone spring, which inherently lacks ideal linearity. Beyond a certain deformation threshold, the silicone’s nonlinear characteristics become markedly pronounced. Furthermore, when the applied force exceeds 0.15 N, the voltage change becomes negligible. At this point, the values of all photosensitive units approach zero, indicating the sensor has reached its maximum measurement range.

(iii) Directional change perception experiment: To verify the sensor’s ability to detect forces from any direction within the plane, this experiment applied a vertical lateral force starting from the orientation corresponding to photosensitive unit 3. The sensor was then continuously rotated counterclockwise while simultaneously recording the voltage values from all four photosensitive units. As shown in the voltage response curve in Fig. 3i, during sensor rotation, the voltages across each photosensitive unit exhibit continuous, coordinated dynamic changes without discrete jumps. During rotation from unit 3 toward unit 1, the voltage of unit 3—initially subjected to external force—gradually declined from its post-loading peak back to its baseline value without external force. It then decreased further with increasing rotation angle, exhibiting a ‘peak-baseline-continuous decay’ gradient characteristic. The voltage of unit 4, adjacent to unit 3, first rises steadily to a local maximum as rotation progresses. It then gradually decreases as the sensor moves away from its corresponding orientation, forming a ‘rise-peak-fallback’ transient response. Furthermore, to demonstrate the sensor’s rapid detection of force direction changes, increasing the rotation speed after 20 s reveals that the rate of change in the voltage response curves of all photosensitive units synchronously accelerates. This exhibits rapid dynamic fluctuations matching the rotation speed, directly confirming the ALL sensor’s excellent dynamic response capability to changes in force direction.

### Velocity estimation experiment

To verify the velocity vector measurement capability of the proposed single sensor unit, no other sensors are used in this section. Under the condition that the ROV body coordinate system is consistent with the world coordinate system, the velocity in the world coordinate system is estimated solely based on data from the ALL sensor. The construction rule of the world coordinate system used in the experiment is shown in Fig. 3c.

It should be noted that all calibration tasks for the ALL sensors in this paper have been pre-completed using the proposed CLANN model. The calibration process employs multiple sets of sample data collected from the ROV under different types of linear motion. Specifically, for the linear motion studied in this section, additional multi-directional linear motion samples were acquired to enhance the model’s generalization capability.

In other studies, performance analysis of the ALL sensors has typically been confined to attitude disturbances within the horizontal plane, such as noise introduced by motions like pitch and roll [9,19,35,36]. However, the impact of multiple disturbances—including vertical oscillations (upward/downward movement) and pitch motions—on sensor measurement performance during two-dimensional planar motion has been less explored. However, such vertical and pitch disturbances alter the relative interaction angle between the sensor and the flow field, posing significant challenges to the velocity perception accuracy of the ALL sensor. Therefore, this subsection and subsequent experiments will validate the effectiveness and robustness of the proposed ALL sensor and fusion algorithm under complex operating conditions that more closely resemble real-world applications, incorporating these composite disturbances.

**Figure 4.**
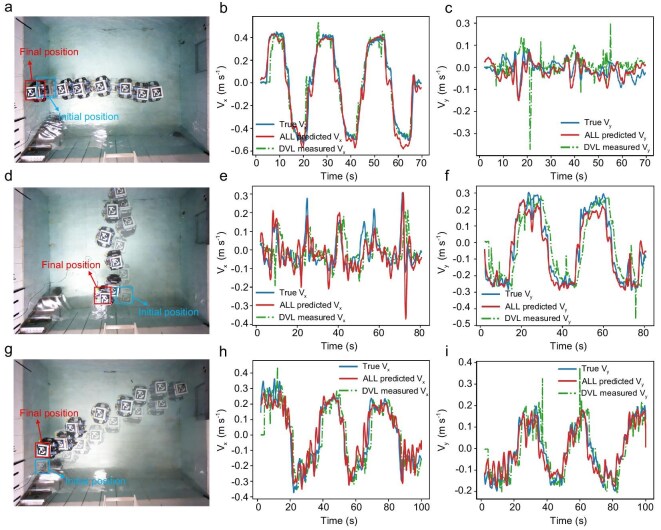
Velocity estimation experiment results. (a) Linear motion along the *x*-axis in the world coordinate system. (b) Predicted velocity in the *x*-direction for linear motion along the *x*-axis in the world coordinate system. (c) Predicted velocity in the *y*-direction for linear motion along the *x*-axis in the world coordinate system. (d) Linear motion along the *y*-axis in the world coordinate system. (e) Predicted velocity in the *x*-direction for linear motion along the *y*-axis in the world coordinate system. (f) Predicted velocity in the *y*-direction for linear motion along the *y*-axis in the world coordinate system. (g) Linear motion in any direction. (h) Predicted velocity in the *x*-direction for linear motion in any direction. (i) Predicted velocity in the *y*-direction for linear motion in any direction.

This section will analyze three typical motion scenarios: linear motion along the *x*-axis of the world coordinate system, linear motion along the *y*-axis of the world coordinate system, and linear motion in any direction. The corresponding motion forms are illustrated in Fig. 4. For simplicity, the *x*-axis and *y*-axis in the world coordinate system are abbreviated as the *x*-axis and *y*-axis in the following sections:

(i) Linear motion along the *x*-axis and *y*-axis: To investigate the velocity vector perception performance of the ALL sensor in underwater environments, this experiment controlled the ROV to perform reciprocating linear motion along the *x*-axis and *y*-axis. A typical motion segment is illustrated in Fig. 4a and d, where the green box represents the ROV’s initial position and the red box indicates its final position. Figure S7 displays the output variation characteristics of all units during the linear motion along the *x*-axis—compared to terrestrial test data, the raw underwater sensor data exhibits significant noise interference. After applying sliding window mean filtering to the raw data, the results are presented as the red dashed line in Fig. S7. When the ROV moves along the *x*-axis, units 1 and 3 exhibit opposite output logic and their variation patterns match the cycle of the ROV’s reciprocating linear motion.

For linear motion along the *x*-axis, Fig. 4b shows the predicted *x*-direction velocity component with a mean absolute error (MAE) of 0.054 m/s and Fig. 4c displays the predicted *y*-direction velocity component with a corresponding MAE of 0.027 m/s. Sequence of *x*- and *y*-direction velocity errors are illustrated in Fig. S8a. Calculations show the MAE for the resultant velocity is 0.05 m/s, with a linearity ($R^2$) of 0.958 for velocity magnitude. For comparison, DVL measurements yield a MAE of 0.047 m/s for the *x*-direction velocity, 0.042 m/s for the *y*-direction velocity, and a MAE of 0.041 m/s for the combined velocity.

The ALL sensor exhibits slightly lower prediction accuracy than the DVL in the *x*-direction but superior accuracy in the *y*-direction, consistent with the experimental data in Fig. 4. Notably, during segments with high-peak signals from small *y*-direction movements, the ALL sensor still achieves good curve fitting. This confirms the sensor’s inherent interference resistance, enabling effective extraction of velocity information from small movements in highly noisy underwater environments.

For linear motion along the *y*-axis, Fig. 4e presents the predicted results for the *x*-direction velocity component, with the MAE of 0.043 m/s. Figure 4f shows the predicted results for the *y*-direction velocity component, corresponding to the MAE of 0.040 m/s. Sequence of *x*- and *y*-direction velocity errors are depicted in Fig. S8b. The MAE for the resultant velocity is 0.043 m/s, with a $R^2$ of 0.941 for velocity amplitude. For comparison, the DVL measurements yielded a MAE of 0.070 m/s for the *x*-direction velocity, 0.079 m/s for the *y*-direction velocity, and 0.070 m/s for the combined velocity. The relevant results are shown in the Table S3.

Comparisons indicate that the ALL sensor generally achieves higher prediction accuracy than the DVL in both the *x* and *y* directions. Figure 4 also reveals that the ALL sensor’s velocity prediction curves for both directions exhibit superior alignment with the camera-derived true values. In contrast, the DVL’s velocity measurement curve displays noticeable spike-like fluctuations. This occurs because the ROV’s motion is not idealized planar motion. Attitude disturbances such as pitch and yaw generated during its movement also affect the DVL. These disturbances alter the beam incidence angle of the DVL transducer, causing distortion in the acoustic echo signals and introducing interference noise into the measurement results.

(ii) Linear motion in any direction: Experiments involving only linear motion along the axis cannot fully validate the sensor’s ability to detect velocity vectors in arbitrary directions. Therefore, while maintaining consistency between the ROV’s body coordinate system and the world coordinate system, a random direction was selected to conduct reciprocating linear motion tests. A typical segment of this motion process is illustrated in Fig. 4g. Figure 4h shows the prediction results for the *x*-direction velocity component, with a MAE of 0.044 m/s; Fig. 4i shows the predicted *y*-direction velocity component with a MAE of 0.027 m/s. Sequence of *x*- and *y*-direction velocity errors is illustrated in Fig. S8c. The MAE for the resultant velocity was 0.038 m/s, with a velocity amplitude $R^2$ of 0.914. For comparison, the DVL measurement yields a MAE of 0.069 m/s for the *x*-direction velocity, 0.043 m/s for the *y*-direction, and a MAE of 0.060 m/s for the combined velocity. The DVL velocity measurement curve exhibits significant high-frequency noise and demonstrates poorer data stability compared to both camera true values and ALL sensor predictions. The relevant results are shown in the Table S3.

### Sensor fusion experiment

The raw data from all sensors represent velocity vectors in the ROV body coordinate system, and their outputs are closely related to the vehicle attitude. When the ROV undergoes attitude changes such as pitching and yawing, the relative pose between the body coordinate system and the world coordinate system shifts. Consequently, the data from the ALL sensor cannot be directly mapped to velocities in the world coordinate system and requires further transformation. To address this issue, this section adopts the proposed CLANN algorithm to fuse data from the IMU and the ALL sensor, enabling prediction of the ROV’s global velocity vector in the world coordinate system. All calibration tasks in this section have been pre-completed using the CLANN model, which leverages multiple sets of sample data collected from the ROV under various motion types, including linear motion, circular motion, and irregular motion.

**Figure 5.**
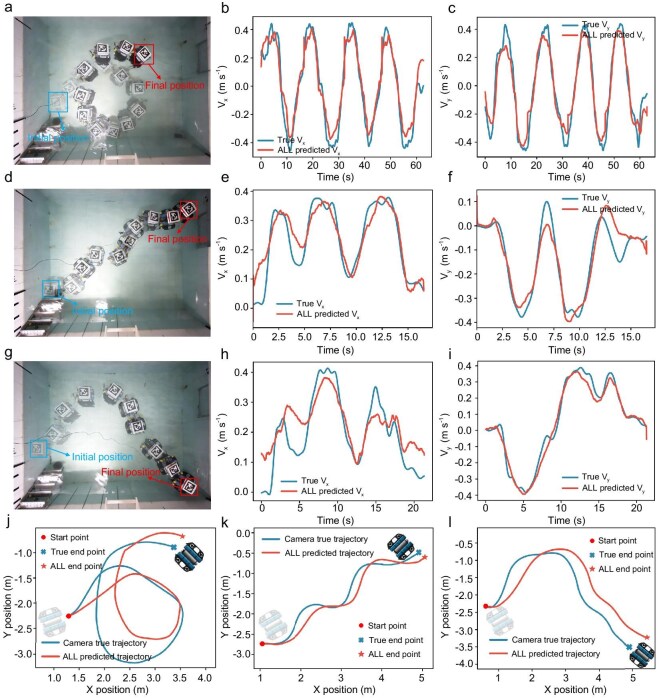
Sensor fusion experiment results. (a) Circular motion. (b) Predicted velocity in the *x*-direction for circular motion. (c) Predicted velocity in the *y*-direction for circular motion. (d) Irregular motion 1. (e) Predicted velocity in the *x*-direction for irregular motion 1. (f) Predicted velocity in the *y*-direction for irregular motion 1. (g) Irregular motion 2. (h) Predicted velocity in the *x*-direction for irregular motion 2. (i) Predicted velocity in the *y*-direction for irregular motion 2. (j) Tracking results for circular motion. (k) Tracking results for irregular motion 1. (l) Tracking results for irregular motion 2.

(i) Circular motion: This experiment controls the ROV to perform circular motion in the world coordinate system, with a typical motion segment selected as shown in Fig. 5a. Figure 5b presents the prediction results for the velocity component in the *x*-direction, with a MAE of 0.068 m/s. Figure 5c shows the prediction results for the velocity component in the *y*-direction, corresponding to a MAE of 0.060 m/s. Sequence of velocity errors are depicted in Fig. S9a. The MAE for the resultant velocity is 0.072 m/s, with a $R^2$ of 0.938 for velocity amplitude.

Based on the fused global velocity vector, the ROV’s motion trajectory was further calculated using the dead reckoning algorithm, with results shown in Fig. 5j. The MAE for position tracking was 0.324 m. The variation of position tracking error as the percentage of total distance is shown in Fig. S10a. Analysis indicates that a single ALL sensor fusing IMU data can effectively capture the overall motion trajectory of the ROV, though deviations persist in local details. Specifically, prediction errors in the velocity vector during peak intervals cause the calculated trajectory’s turning radius to be smaller than the actual trajectory, leading to cumulative trajectory errors.

(ii) Irregular motion: The aforementioned experiments focused on studying regular motion. This experiment selected two segments of irregular motion to analyze the velocity and trajectory prediction of the ROV, as shown in Fig. 5d and g. The prediction results and sequence of velocity errors are shown in Fig. 5 and Fig. S9. For irregular motion 1: the MAE for the *x*-direction velocity component is 0.035 m/s. The MAE for the *y*-direction velocity component is 0.037 m/s. The MAE for the resultant velocity is 0.030 m/s. The velocity amplitude $R^2$ is 0.885. For irregular motion 2: the MAE for the *x*-direction velocity component is 0.070 m/s. The MAE for the *y*-direction velocity component is 0.043 m/s. The MAE for the resultant velocity is 0.050 m/s and the velocity amplitude $R^2$ is 0.822.

Furthermore, based on the experiments conducted in this paper, it can be concluded that the lateral line sensor proposed herein can operate within the range of 0–0.4 m/s.

Further calculations yielded the ROV’s motion trajectory, with results shown in Fig. 5k and l. The MAE for position tracking during irregular motion 1 was 0.1614 m, while that for irregular motion 2 was 0.3664 m. The variation of position tracking error as the percentage of total distance is shown in Fig. S10b and c. Finally, the relevant data for the aforementioned different motions are summarized in Table S4.

### Future work and application

In our future work, we will focus on two key areas: iterative optimization of core performance and research into application-specific challenges. Given the room for improvement in the accuracy of current sensors, we will address this issue from both algorithmic and mechanical perspectives by refining the sensor’s mechanical structure and optimizing the perception algorithms to further enhance the measurement accuracy of the proposed lateral line sensor. To address the self-localization challenges currently faced by biomimetic robotic fish, we aim to successfully integrate the flexible lateral line sensor developed in this study with a biomimetic robotic fish platform. Leveraging the robotic fish’s low-disturbance swimming characteristics, we will conduct multi-scenario, full-condition validation experiments in natural water bodies under varying flow velocities and aquatic environments. By combining this sensor with other auxiliary sensors, we will construct a wide-area flow field distribution model of the target water body. Based on the pre-established prior information of the wide-area flow field, we aim to ultimately achieve long-term, precise autonomous positioning of the robotic fish in complex water bodies even under scenarios where velocity sensors such as the DVL fail.

## CONCLUSIONS

Inspired by the lateral line system in fish, this paper proposes a flexible ALL sensor unit based on optical flux for measuring the magnitude and direction of flow velocity. Subsequently, physical modeling and FSI simulations analyze the sensor’s force response and deformation characteristics. To achieve sensor calibration, a hybrid neural network algorithm named CLANN is introduced. This algorithm models and calibrates the ALL sensor unit in underwater environments, enabling flow velocity estimation and multi-sensor fusion. It is also applicable to modeling tasks for other flexible sensors. Finally, the sensor was installed on an ROV for experimental validation, focusing on evaluating the impact of disturbances such as pitch and yaw during planar motion to verify the effectiveness of the sensor and algorithm. Experimental results indicate that using this sensor unit and neural network algorithm yields a MAE of 0.055 m/s for velocity measurement along the *x*-axis and 0.043 m/s along the *y*-axis. The MAE for velocity magnitude reached 0.048 m/s, while the directional MAE was 16.49°. The $R^2$ value reached 0.896, and the trajectory prediction MAE was 0.284 m.

## Supplementary Material

nwag337_Supplemental_Files
