# Supplementary material for: Flexible artificial lateral line based on luminous flux for underwater velocity vector estimation
Source: Natl Sci Rev. 2026 Jun 5;13(14):nwag337. doi: 10.1093/nsr/nwag337 (PMC13348251; doi:10.1093/nsr/nwag337)
Supplement: nwag337_Supplemental_Files [file nwag337_supplemental_files.zip › supplementary file_Flexible Artificial Lateral Line Based on Luminous Flux for Underwater Velocity Vector Estimation.pdf]

Supplementary Materials for

Flexible Artificial Lateral Line Based on Luminous Flux for Underwater Velocity Vector Estimation

Xintao Wang[1,2,†], Zhengwei Li[2,†], Zhuoliang Zhang[1,†], Junfeng Fan[1,†], Yaming Ou[1,2], Xiangyu Sun[1,2], Min Tan[1,*], Long Cheng[2,*], Chao Zhou[1,*]

[1] Laboratory of Cognition and Decision Intelligence for Complex Systems, Institute of Automation, Chinese Academy of Sciences, Beijing 100190, China

[2] School of Artificial Intelligence, University of Chinese Academy of Sciences, Beijing 100049, China

[*] Corresponding authors. Email: min.tan@ia.ac.cn; long.cheng@ia.ac.cn; chao.zhou@ia.ac.cn

Contents

Supplementary Notes

Supplementary Tables

Supplementary Figures

Supplementary Videos

Supplementary References

Supplementary Notes

Supplementary Note 1. The ALL sensor physical model

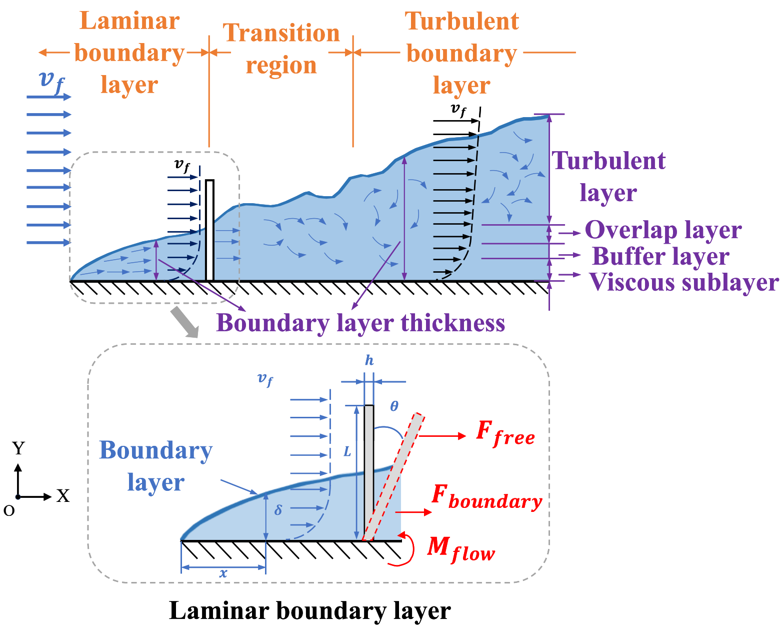

Supplementary Figure 1. Laminar boundary layer near the ALLs. Within the boundary layer, the flow velocity gradually increases from zero to the free-stream velocity v_f.

This paper employs boundary layer theory to model the proposed ALL sensor. Boundary layer theory divides fluid flow into a boundary layer region and a main stream region. The boundary layer region refers to a thin layer of fluid near a solid surface. Within the boundary layer, fluid velocity gradually increases from zero velocity at the wall (no-slip condition) to the main-stream velocity (99% of free-flow velocity velocity)[1], as shown in Fig. S1.

The boundary layer can be broadly categorized into laminar boundary layer, transition region, and turbulent boundary layer based on different flow conditions. To facilitate fluid dynamics analysis of the sensor under varying flow velocities, this paper focuses on the laminar boundary layer context.

For the boundary layer region, its thickness satisfies the following formula:

$$\delta = \frac{5}{\sqrt{Re_x}} x \tag{S1}$$

where Re_x is the local Reynolds number, satisfying the Reynolds number formula $Re_x = \rho v_f x / \mu$. ρ is the water density. v_f is the main-stream velocity. μ is the dynamic viscosity. x denotes the distance between the current position and the leading edge.

Based on this, the fluid velocities v in the boundary layer region and the main stream region can be obtained:

$$v = \begin{cases} v_f & \text{if } y > \delta \\ v_f \cdot \left(\frac{2y}{\delta} - \frac{y^2}{\delta^2} \right) & \text{if } 0 < y < \delta \end{cases} \tag{S2}$$

Here, y denotes the perpendicular distance relative to the solid surface.

The ALL sensor employs a cross-shaped rocker. Since the thickness of the cross is significantly smaller than its width, the influence of rocker's thickness is neglected for analytical and computational simplicity. The rocker thus comprises two mutually perpendicular primary inflow surfaces, as shown in Fig. S2(a). The sensor reaches equilibrium under water flow impact. At this point, the sensor's rocker is primarily subjected to the fluid force F_{flow} and the elastic force of its own silicone spring F_e, satisfying Newton's Second Law $F_{flow} = F_e$.

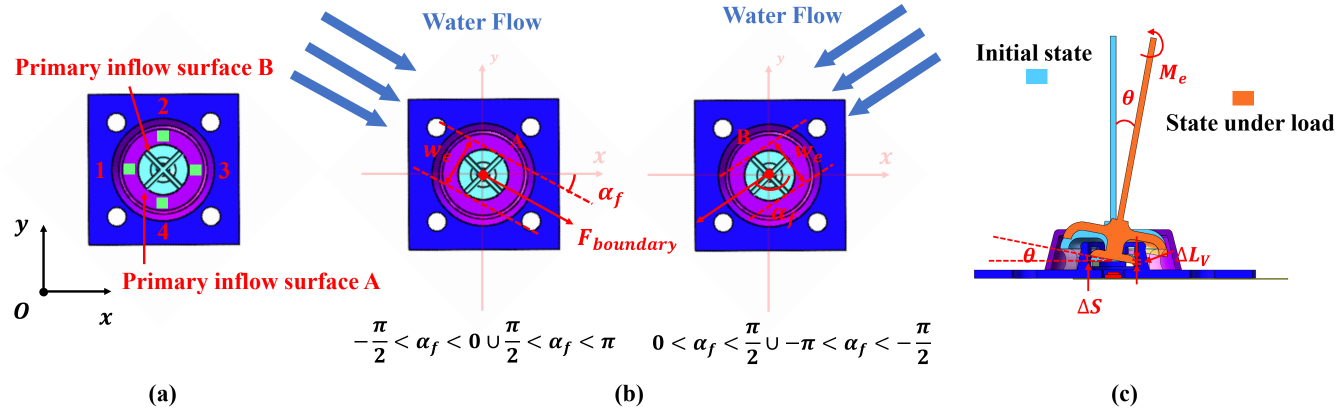

Supplementary Figure 2. Analysis diagram of the ALL sensor model. (a) Position of the sensor's photosensitive units and primary inflow surfaces, along with the coordinate system rules. (b) Discussion on equivalent surface width for different flow directions. (c) Sensor deformation analysis.

The fluid forces acting on the sensor primarily consist of two components: boundary layer fluid forces $F_{boundary}$ and main-stream fluid forces F_{free}, which are related by the following equation:

$$F_{flow} = F_{boundary} + F_{free} \tag{S3}$$

Fig. S2(a) shows photosensitive units 1–4 (green squares) and the two primary inflow surfaces A and B. Establish a coordinate system as shown in Fig. S2(a), with the direction of photosensitive element 3 as the positive x-axis, the direction of photosensitive element 2 as the positive y-axis, and the z-axis direction determined by the right-hand rule.

The two primary inflow surfaces form angles of 45° with the x and y axes, respectively. Assume the water flow strikes the rocker at an angle α_f relative to the positive x-axis direction, where clockwise rotation in the OXY plane is defined as positive. At this point, both primary inflow surfaces of the cross will experience fluid forces, and $F_{boundary}$ should be composed of multiple component forces. Since pressure difference resistance is much greater than friction resistance, friction resistance can be neglected, and only pressure difference resistance is considered. Here, the fluid force is calculated using the equivalent inflow surface method, as shown in Fig. S2(b).

The angle requires case-by-case analysis. When $-\frac{\pi}{2} < \alpha_f < 0$ and $\frac{\pi}{2} < \alpha_f < \pi$, the primary inflow surface A serves as the maximum primary inflow surface, and the equivalent surface width w_e is:

$$w_e = w \cdot |\sin(\alpha_f - \frac{\pi}{4})| \tag{S4}$$

When $0 < \alpha_f < \frac{\pi}{2}$ and $-\pi < \alpha_f < -\frac{\pi}{2}$, the primary inflow surface B serves as the maximum primary inflow surface. Similarly, the formula for calculating the equivalent surface width is derived as follows:

$$w_e = w \cdot |\sin(\alpha_f + \frac{\pi}{4})| \tag{S5}$$

The formula for calculating $F_{boundary}$ and F_{free} is as follows:

$$\begin{aligned} F_{\text{boundary}} &= \int_0^\delta \mathrm{d}F_{boundary}(y) \\ &= \int_0^\delta \frac{1}{2}\rho C_D v^2 w_e \mathrm{d}y \\ &= \frac{1}{2}\rho C_D w_e v_f^2 \int_0^\delta (\frac{2y}{\delta} - \frac{y^2}{\delta^2})^2 \mathrm{d}y \end{aligned} \tag{S6}$$

where C_D is the drag coefficient, w is the width of the cross's primary inflow surface.

$$
\begin{aligned}
F_{\text{free}} &= \int_{\delta}^{L} \mathrm{d}F_{\text{free}}(y) \\
&= \int_{\delta}^{L} \frac{1}{2}\rho C_D v^2 w_e \mathrm{d}y \\
&= \frac{1}{2}\rho C_D w_e v_f^2 (L-\delta)
\end{aligned}
\tag{S7}
$$

where L is the length of the cross-shaped rocker. Combining equations (S6) and (S7) yields the fluid force acting on the sensor:

$$
\begin{aligned}
F_{flow} &= \frac{1}{2}\rho C_D w_e v_f^2 \int_0^{\delta} (\frac{2y}{\delta} - \frac{y^2}{\delta^2})^2 \mathrm{d}y + \\
&\quad \frac{1}{2}\rho C_D w_e v_f^2 (L-\delta) \\
&= \frac{1}{2}\rho C_D w_e v_f^2 [\int_0^{\delta} (\frac{2y}{\delta} - \frac{y^2}{\delta^2})^2 \mathrm{d}y + (L-\delta)]
\end{aligned}
\tag{S8}
$$

Therefore, the fluid torque M_{flow} is calculated as follows:

$$
\begin{aligned}
M_{flow} &= \int_0^{Lcos\theta} y \mathrm{d}F_{flow}(y) \\
&= \frac{1}{2}\rho C_D w_e v_f^2 \cos^2\theta \cdot \\
&\quad [\int_0^{\delta} (\frac{2y}{\delta} - \frac{y^2}{\delta^2})^2 y \mathrm{d}y + \int_{\delta}^{L} y \mathrm{d}y] \\
&= \frac{1}{2}\rho C_D w_e v_f^2 \cos^2\theta \cdot \\
&\quad [\int_0^{\delta} (\frac{2y}{\delta} - \frac{y^2}{\delta^2})^2 y \mathrm{d}y + \frac{L^2-\delta^2}{2}]
\end{aligned}
\tag{S9}
$$

θ is the inclination of the rocker relative to its initial vertical position.

The rocker of the ALL sensor is made of rigid material, while the outer layer of the rocker is connected to silicone spring, which can be simplified as a spring-damping system[2]. Therefore, the relationship between the sensor tilt angle θ and the elastic torque of the silicone spring M_e can be derived using the rigid body rotation equation about a fixed axis:

$$
M_e = K \cdot \theta + C \cdot \frac{\mathrm{d}^2\theta}{\mathrm{d}t} + J \cdot \frac{\mathrm{d}^2\theta}{\mathrm{d}t^2}
\tag{S10}
$$

In the equation, K represents the elastic torsional stiffness restoring the rocker to its initial vertical position. C denotes the viscous damping coefficient, and J indicates the moment of inertia. Considering the sensor reaching a steady state under water flow impact, the angular velocity $\dot{\theta}$ and angular acceleration $\ddot{\theta}$ can be neglected. The torque of the fluid balances the elastic torque of the silicone spring, i.e., $M_e = M_{flow}$. Thus, we obtain:

$$
\begin{aligned}
K \cdot \theta &= \frac{1}{2}\rho C_D w_e v_f^2 \cos^2\theta \cdot \\
&\quad [\int_0^{\delta} (\frac{2y}{\delta} - \frac{y^2}{\delta^2})^2 y \mathrm{d}y + \frac{L^2-\delta^2}{2}]
\end{aligned}
\tag{S11}
$$

Due to the short propagation path and minimal interference, attenuation during light transmission can be neglected. Under ideal initial conditions, the inner layer of the sensor contact should partially block the photosensitive unit, as shown in Fig. S2(c). When force is applied in a certain direction, the rocker tilts toward that direction, reducing the light flux reaching the photosensitive unit. Given the small dimensions of the sensor, when water flows strike it from any direction, the arc motion of the inner layer during rocker tilt can be approximated as linear

motion, i.e., $\Delta L_V = \Delta S$. Here, ΔL_V represents the change in the area of the photosensitive unit obscured relative to the initial state. ΔS denotes the change in the arc length rotated by the inner layer of the rocker, satisfying $\Delta S = r_d \cdot \Delta\theta$. r_d is the radius of the rocker's inner layer. The tilt of the contact causes a linear change in the luminous flux, described by the following relationship:

$$\Phi_V = \Phi_{V0} - K_s \cdot \Delta S \tag{S12}$$

Here, Φ_V denotes the luminous flux of the photosensitive unit, Φ_{V0} denotes the luminous flux of the photosensitive unit in its initial state, K_s denotes the proportionality coefficient between the change in luminous flux and the change in arc length, and i denotes the ith photosensitive unit.

The relationship between the output voltage of the photosensitive unit V_{out} and the luminous flux Φ_V is as follows:

$$\begin{aligned} V_{\text{out}} &= I_{\text{out}} \cdot R_L \\ &= \left[K_V \cdot \Phi_V \cdot \beta(\theta_V) + I_{\text{DARK}}\right] \\ &\quad \cdot \left[1 + \gamma(T)\right] \cdot R_L \end{aligned} \tag{S13}$$

In the equation, I_{out} is the output current of the photosensitive unit. R_L is the load resistance, and K_V is the sensitivity coefficient. β represents the angular response coefficient, which is related to the incident angle θ_V. I_{DARK} is the dark current, and γ is the temperature compensation factor, which is related to temperature T.

The aforementioned formula relating the tilt angle to the voltage value applies only to a specific photosensitive unit. When water strikes the sensor from any direction, the sensor tilts along the flow direction, causing voltage changes across multiple photosensitive units simultaneously. The tilt angle thus possesses components along both the x and y axes. Assume the rods at the upper end of the contact point are projected onto the OXZ and OYZ planes, forming angles θ_x and θ_y with the z-axis, respectively, satisfying the following relationship:

$$\theta = |\vec{\theta}| = \sqrt{\theta_x^2 + \theta_y^2} \tag{S14}$$

$$\frac{\theta_y}{\theta_x} = |\tan \alpha_f| \tag{S15}$$

Since the above equation can only calculate the absolute value of $\tan \alpha_f$, α_f can take multiple values. Precise value of α_f must be determined by analyzing the specific variation patterns of voltages across units 1 through 4. Furthermore, this equation S15 applies only when $\alpha_f \neq \frac{\pi}{2} \cup -\frac{\pi}{2}$.

Therefore, combining the above formula(S11)-(S15) yields the following system of equations:

$$\begin{cases} K \cdot \theta = \dfrac{1}{2}\rho C_D w_e v_f^2 \cos^2\theta \cdot \\ \qquad \left[\displaystyle\int_0^\delta \left(\frac{2y}{\delta} - \frac{y^2}{\delta^2}\right)^2 y\,\mathrm{d}y + \frac{L^2 - \delta^2}{2}\right] \\ \overrightarrow{V_{\text{out}}} = \left[K_r \cdot \overrightarrow{\Phi_V} \cdot \beta(\theta_V) + I_{\text{DARK}}\right] \\ \qquad \cdot \left[1 + \gamma(T)\right] \cdot R_L \\ \Phi_{V_x} = \Phi_{V0_x} - K_s \cdot r_d \cdot \theta_x, \\ \Phi_{V_y} = \Phi_{V0_y} - K_s \cdot r_d \cdot \theta_y, \\ \theta = |\vec{\theta}| \end{cases} \tag{S16}$$

In the equations, $\overrightarrow{V_{\text{out}}} = V_{\text{out}_x} \cdot \vec{x} + V_{\text{out}_y} \cdot \vec{y}$. V_{out_x} and Φ_{V_x} represent the output voltage and luminous flux of the photosensitive unit with a corresponding decrease in voltage value along the x-axis, respectively. V_{out_y} and Φ_{V_y} represent the output voltage and luminous flux of the photosensitive unit with a corresponding decrease in voltage value along the y-axis. By solving equation (S16), the magnitude v_f and direction α_f of the flow velocity can be determined.

Supplementary Note 2. FSI-based simulation analysis for the ALL sensor

To further analyze the impact of water flow on sensor deformation, this paper simulates the sensor based on FSI analysis. The simulation conditions are set as shown in Fig. S3. The fluid domain adopts a cylindrical design, with the sensor positioned at the center of the fluid domain and fixed at the bottom to prevent displacement caused by fluid forces. The solid domain encompasses the entire sensor, with each module parameterized according to its material properties. Due to the presence of internal cavity, a solid model of the cavity was also created and assigned air material property to ensure simulation accuracy. At the inlet, flow velocities ranging from 0.05 m/s to 0.8 m/s were applied in 0.05 m/s increments to obtain the deformation characteristics of the solid structure (as shown in Fig.S4 and Fig.S5). It should be noted that the deformation in Fig.S4 and Fig.S5 refers to the displacement of the sensor relative to its initial state.

Due to the rigid material of the rocker, when water flow impacts the sensor contacts, the silicone spring deforms, causing the entire contact assembly to tilt. The tilt direction aligns with the water flow direction, consistent with the sensor's principle. Structural deformation is concentrated in the upper flow-exposed region, while the base deformation approaches zero, reflecting the localized feature of water flow loading. As flow velocity increases, the maximum structural deformation exhibits a significant growth trend: at v=0.3 m/s, the maximum deformation is 0.294 mm; at v=0.5 m/s, it increases to 0.81 mm; and at v=0.8 m/s, the maximum deformation rises to 2.045 mm.

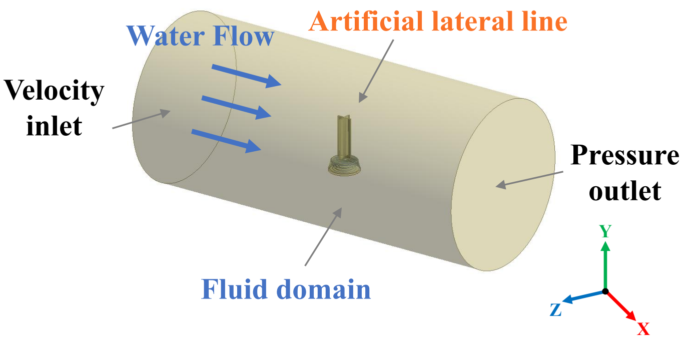

Supplementary Figure 3. Setup Diagram of FSI Simulation. A cylindrical fluid domain is employed, with the fluid direction rotating counterclockwise by 45° along the X-axis.

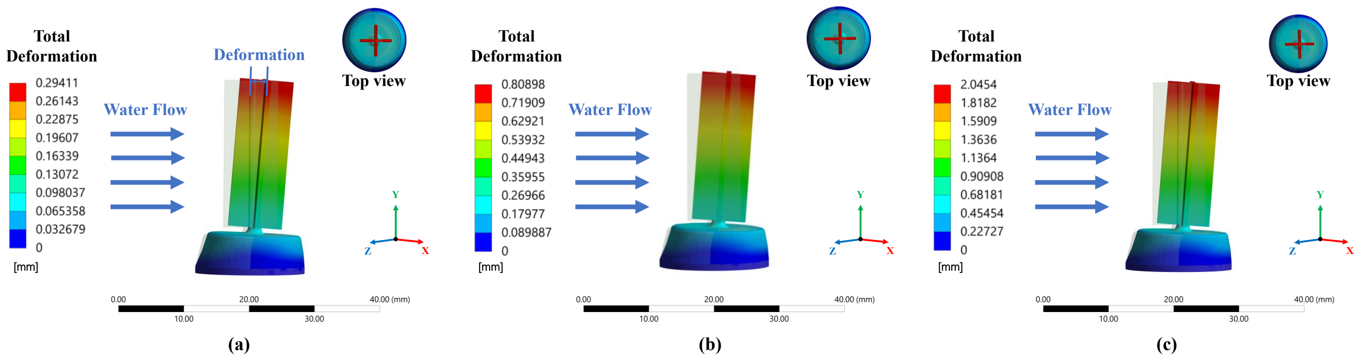

Supplementary Figure 4. FSI Simulation Results. Deformation refers to the displacement of the sensor relative to its initial state. (a) Total deformation at a flow velocity of 0.3 m/s. (b) Total deformation at a flow velocity of 0.5 m/s. (c) Total deformation at a flow velocity of 0.8 m/s.

As shown in the Fig.S5, the sensor deformation exhibits a nonlinear trend as the flow velocity increases. The tilt angle θ is calculated using the ratio of the deformation amount within the OXZ plane measured by the sensor to the length of the rocker. Calculations show that $\theta/\cos^2\theta$ is proportional to the square of the flow velocity v_f, and the proportionality constant is found to be $k = 0.000164$. This verifies the validity of the proposed physical model.

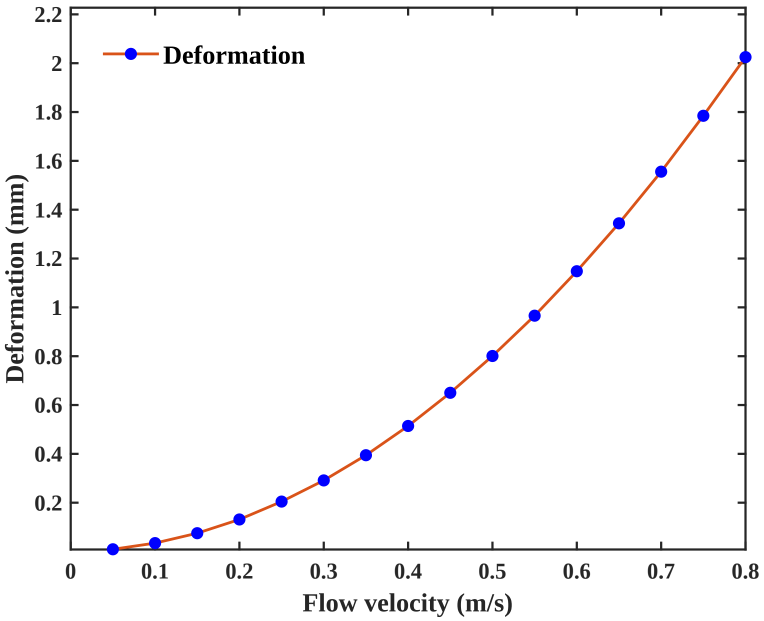

Supplementary Figure 5. Sensor deformation at different flow velocities.

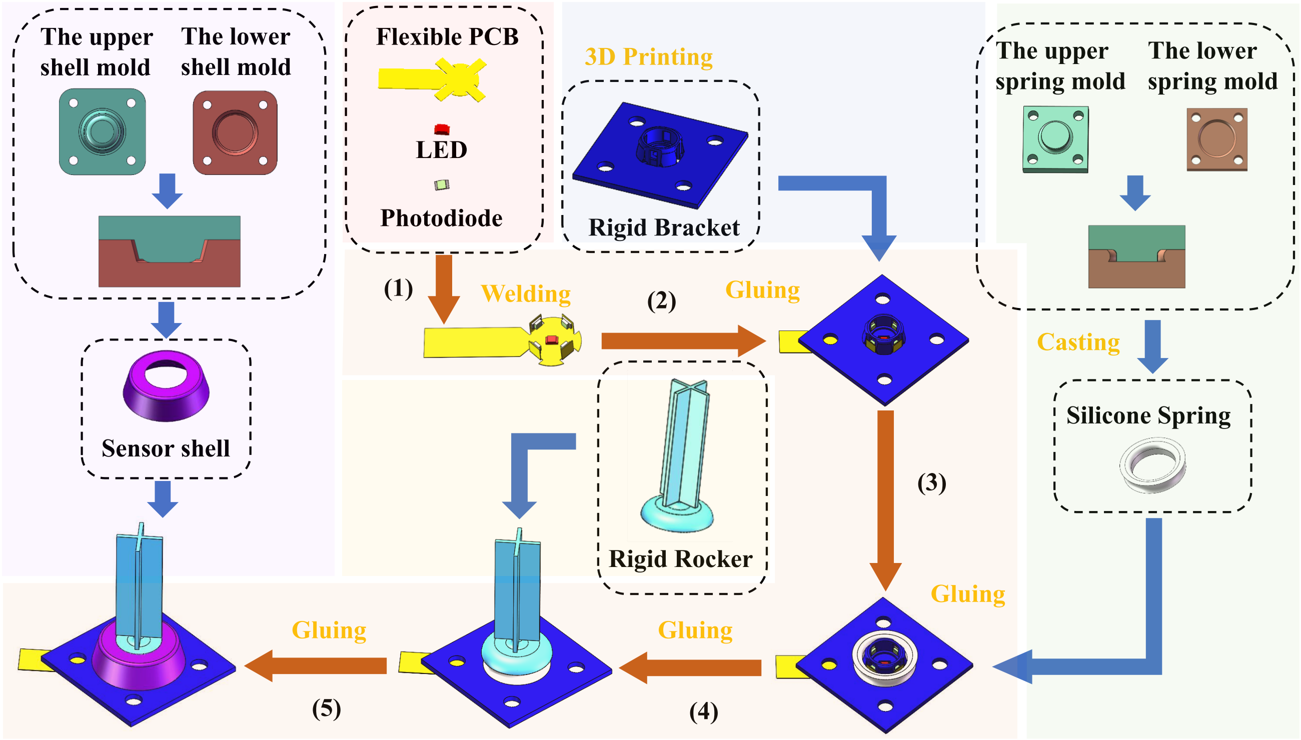

Supplementary Figure 6. Fabrication process and steps for the ALL sensor. The red arrows indicate key assembly steps: the rigid bracket and rigid rocker are 3D printed, while the silicone spring and flexible shell are produced via injection molding. The mold is fabricated using 3D printing.

Supplementary Note 3. Sensor fabrication

The fabrication of the ALL sensor was completed following the process shown in Fig.S6(a), primarily comprising the following four steps: 1) Fabrication of the flexible shell: The shell was produced using an injection molding method, which first required preparing the mold. The shell mold consists of upper and lower halves, with the shell mold design shown in Fig.S6(a). All molds were fabricated via 3D printing. Ecoflex00-30 platinum-cured silicone was used. Part A, part B and black pigment were mixed in a beaker at a ratio of 1:1:0.1, thoroughly blended, then placed in a vacuum chamber to eliminate air bubbles. Parts A and B primarily form the silicone matrix, while the black pigment provides opacity to eliminate interference from external stray light on sensor performance. The mixed silicone was poured into the shell mold and left at room temperature until fully cured. The cured silicone was then demolded to retrieve the shell. 2) Silicone spring fabrication: The manufacturing process is similar to the shell. Silicone spring is also produced via injection molding. All spring molds are manufactured using 3D printing. The silicone spring is made from the same silicone material as the housing. Mix part A and B in a 1:1 ratio until uniform, then eliminate air bubbles before injecting into the mold. Cure using the same method as for the shell. 3) Bracket, rocker and FPCB fabrication: Both the bracket and rocker are 3D printed. The FPCB is manufactured from printed designs, with LEDs and photosensitive components soldered onto its surface. 4) Sensor assembly: As indicated by the red arrow in Fig.S6(a), first bend the four corners of the soldered FPCB and insert them into the bracket's grooves, securing them with instant adhesive. Next, using Valigoo V-1510 silicone-specific flexible adhesive, bond the silicone spring, bracket and rocker in sequence. Finally, install the shell, apply adhesive for sealing, and complete the sensor assembly. The physical sensor is shown in Fig.1(e).

Photosensitive unit

The photosensitive unit selected for this paper is the APDS-9005 low-cost analog-output ambient light sensor developed by Broadcom, which features a miniature chip LED in a lead-free surface-mount package. The sensor measures $1.5mm1.6mm0.55mm$, operates at a voltage range of $1.8V$ to $5.5V$, has an absolute maximum voltage rating of $6V$, and operates within a temperature range of $-40°C$ to $+85°C$. It supports a light reception cone angle of±35°. It contains a photodetector with a spectral response close to that of the CIE standard photopic observer (similar to the human eye), with a peak sensitivity at a wavelength of $500nm$. The sensor uses an analog current output with linear output across a wide illuminance range and minimal sensitivity deviation under different light sources. At an ambient temperature of $25°C$ and with an external load resistance $R_L = 1k\Omega$, the rise/fall time of the photosensitive element is $5ms$, and the settling time (i.e., the time required for the sensor to stabilize at 95% of the accurate output value after power-on) is 10 ms.

LED

The LED selected for this paper is the HL-PSC-2012S9AC model from HONGLITRONIC. This LED features a colorless transparent lens and emits red light. The LED has a luminous intensity of $140mcd$, a power consumption of $72mW$, and a beam angle of 140°. It has a forward voltage of 2.4 V, a forward current of $20mA$, a wavelength range of $626nm$ to $636nm$, and an operating temperature range of $-40°C$ to $+85°C$.

Supplementary Note 4. Sensor data preprocessing

The raw voltage signals from the photosensitive units contain 50 Hz power-line interference, high-frequency sensor noise, and complex underwater disturbance noise. We employ adaptive windowed averaging filtering to preprocess the raw sensor data.

For a sequence of length N, standard moving average filtering can introduce distortion at the beginning and end of the sequence (where $i \leq m$ or $i > N - m$) due to insufficient data. This article uses edge padding to solve this problem.

$$y'_k = \begin{cases} y_1, & k \leq 0 \\ y_k, & 1 \leq k \leq N \\ y_N, & k \geq N \end{cases} \tag{S17}$$

y_1 denotes the first value in the original data sequence, and y_N denotes the last value in the original data sequence. y'_k denotes the sensor measurement at the k-th sampling time.

Then, adaptive sliding window mean filtering is applied to the 4-channel ALL sensor voltage signal sequence after interpolation.

$$\hat{y}_i = \frac{1}{w} \sum_{k=i-m}^{i+m} y'_k \tag{S18}$$

Here, $\hat{y}_i$ denotes the filtered output at the i-th time step, w is the window size, and m is the window half-width, i.e., the number of data points taken on either side of each midpoint.

The specific dimensions for window w are:

$$w = \begin{cases} 3, & \left\lfloor \frac{N}{20} \right\rfloor < 3 \\ \left\lfloor \frac{N}{20} \right\rfloor, & 3 \leq \left\lfloor \frac{N}{20} \right\rfloor \leq 50 \text{ and odd} \\ \left\lfloor \frac{N}{20} \right\rfloor + 1, & 3 \leq \left\lfloor \frac{N}{20} \right\rfloor \leq 50 \text{ and even} \\ 51, & \left\lfloor \frac{N}{20} \right\rfloor > 50 \end{cases} \tag{S19}$$

In addition, before feeding the data into the neural network, we performed Min-Max normalization on all data. We estimated the global minimum and maximum values from the training set, scaled the inputs to the range of 0 to 1 before training and testing, and denormalized the flow velocity results after prediction. The purpose of this was to unify the scales of multi-source signals and ensure compatibility with the autoencoder architecture.

$$\hat{x} = \frac{x - x_{\min}}{x_{\max} - x_{\min}} \tag{S20}$$

Here, $\hat{x}$ represents the normalized result, with a range of 0 to 1. x denotes the raw data, including the voltage from the side-line sensors and the quaternion representing the IMU attitude. x_{min} is the global minimum value for this column in the training set, and x_{max} is the global maximum value for this column in the training set.

Supplementary Tables

Supplementary Table 1. Structural Parameters of the Sensor

Structure	Diameter (mm)	Height (mm)
Rigid bracket	8	4.3
FPCB core section	9.52	0.1
Shell	16.34	4.81
Silicone spring	12.62	2.5
Rigid rocker	12.62	26.81
Sensor	16.34	28.91

Due to the irregular structures of individual sensor modules, the table indicates the maximum dimensions for the entire sensor and each module, excluding extended sections of the FPCB and base.

Supplementary Table 2. Parameters of the Model

Network	Module	Parameters
FN	Encoder(Conv1)	(8,16,3)
	Encoder(Conv2)	(16,32,3)
	Decoder(TransConv1)	(32,16,3)
	Decoder(TransConv2)	(16,8,3)
BN	LSTM	(64,1)
	ResNet	(32,64)
	Causal Self-Attention	(64,2,1)
LFN	FC Layer	(64,2)

The encoder module comprises two convolutional layers with parameters (8,16,3) and (16,32,3), respectively. The first two numbers represent the input and output dimensions of each convolutional layer, while the last digit denotes the size of the convolutional kernel. The decoder comprises two deconvolution layers with parameters mirroring those of the encoder, where positional parameters retain identical meanings. The LSTM's (64,1) denotes a hidden layer dimension of 64 and a layer count of 1. The ResNet's (32,64) indicates input and output dimensions of 32 and 64, respectively. The causal self-attention has parameters (64,2,1), where the input and output dimensions are identical at 64. This self-attention features two attention heads and a single attention block. The fully connected layer has an input dimension of 64 and an output dimension of 2.

The sensor's four-channel voltage values and the IMU attitude quaternion are input into the feature network, where an encoder extracts them into a 32-dimensional feature vector. This feature vector is transmitted via two paths: one path passes through a decoder to reconstruct the 8-dimensional original input data and feed it into the loss function network, while the other path inputs into the backbone network to obtain a 64-dimensional output feature. The LSTM and ResNet each receive the output from the encoder module of DAE. Their results are fused and fed into a causal self-attention. The causal self-attention's output is then input into the loss function network. This network maps the 64-dimensional feature data from the backbone network into 2-dimensional velocity

components via a fully connected layer, using the result for loss function calculation.

It should be noted that the input dimensions of the model are adjustable. The velocity vector in the body coordinate system can be directly predicted by inputting the four voltage values from the ALL sensor.

Supplementary Table 3. The Performance of ALL Sensor and IMU in Different Experiments

Different experiments	Linear motion along x-axis	Linear motion along y-axis	Linear motion in any direction
MAE of Vx (ALL)	0.054 m/s	0.043 m/s	0.044 m/s
MAE of Vx (DVL)	0.047 m/s	0.070 m/s	0.069 m/s
MAE of Vy (ALL)	0.027 m/s	0.040 m/s	0.027 m/s
MAE of Vy (DVL)	0.042 m/s	0.079 m/s	0.043 m/s
MAE of Velocity (ALL)	0.043 m/s	0.043 m/s	0.038 m/s
MAE of Velocity (DVL)	0.041 m/s	0.070 m/s	0.060 m/s

Supplementary Table 4. the Performance of All in Different Experiments

Different experiments	All experiments	Straight trajectory	Circular trajectory	Random trajectory1	Random trajectory2
Linearity (R^2)	0.896	0.938	0.938	0.885	0.822
MAE of Vx	0.055m/s	0.047 m/s	0.068 m/s	0.035 m/s	0.070 m/s
MAE of Vy	0.043m/s	0.031 m/s	0.060 m/s	0.037 m/s	0.043 m/s
MAE of Velocity	0.048m/s	0.041 m/s	0.072 m/s	0.030 m/s	0.050 m/s
MAE of direction	16.49°	26.91°	14.24°	8.29°	16.54°
Position tracking error	0.284m	/	0.324m	0.1614m	0.3664m

Supplementary Table 5. Comparison Table of Sensor Performance

**Sensor**	**Principle**	**Measurement**	**Platform**	**Numbers**	**Advantages**	**Limitations**
Zhang et al.[3]	Strain	Range: 0-0.4m/s<br>Speed MAE:0.018m/s<br>Direction MAE: 10.6°	Robotic fish	8	Hardware-level noise reduction, high precision	An array is required; a single unit lacks vector capabilities
Zhang et al.[4]	Vision	Range: 0.325-0.715m/s<br>Speed MAE: 0.0119 m/s<br>Direction MAE: 3.54°	Robotic fish	1	Single-unit vector, high precision	Low-flow dead zones (<0.325m/s); bulky
He et al.[5]	Magnetic	Range: 0-0.4m/s<br>Speed MAE: 0.0152 m/s (straight); 0.0125m/s (turn)<br>Direction MAE: /	Robotic fish	3	3D Decoupling, Resistance to Self-Motion noise	Requires an array; direction-dependent on external geomagnetic sensors
Tian et al.[6]	Fiber	Range: 0.037-0.072 m/s<br>Speed MAE: /<br>Direction MAE: /	Fixed	1	High sensitivity, resistant to electromagnetic interference	Narrow measurement range, slow response
Zheng et al.[7]	Pressure	Range: 0.05-0.25m/s<br>Speed MAE: /<br>Direction MAE: 14.4°	Robotic fish	11	Online Estimation of 3D Multi-Motion States for Freely Swimming Robotic Fish	Requires an array
Ours	Luminous flux	**Range: 0-0.4m/s**<br>Speed MAE: 0.048m/s<br>Direction MAE: 16.49°	**ROV**	**1**	**Single-unit omnidirectional vector sensing with no low-flow dead zones and Compact size**	The absolute accuracy is slightly lower

Supplementary Table 6. Composition of the Dataset

Data types	Duration(h)	Percentage(%)	Note
Linear motion along the x-axis	0.2236	19.18	Covering the entire range of flow velocities from 0 to 0.4 m/s along the x-axis of the global coordinate system
Linear motion along the y-axis	0.2686	23.04	Covering the entire range of flow velocities from 0 to 0.4 m/s along the y-axis of the global coordinate system
Linear motion in any direction	0.3358	28.80	Linear motion in any direction under a fixed ROV coordinate system
Circular motion	0.275	23.58	Circular motion with various radii
Irregular motion	0.063	5.40	Including complex motion trajectories such as S-curves and random walks

A total of 1.166 hours of continuous multi-sensor data has been collected in this study, which covers comprehensive operating scenarios of the underwater robot under diverse flow velocities, motion patterns, and environmental conditions. Detailed information of the dataset is presented in Table S6. The dataset is partitioned into the training set, validation set, and test set in chronological order to strictly avoid data leakage. The duration of the test set is 0.1172 hours, accounting for 10.05% of the total dataset. The remaining data is divided into the training set and validation set at a ratio of 7:3. Since all data from the lateral line sensors are upsampled to 30 Hz, the training set consists of 57 groups of time series with a total of 114,600 time steps.

Supplementary Figures

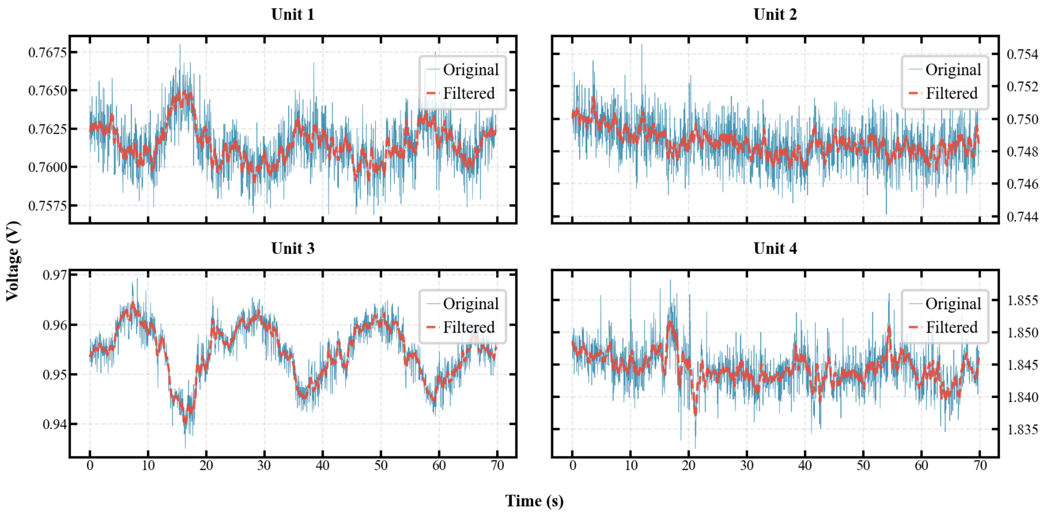

Supplementary Figure 7. Raw data from all sensors' photosensitive units. The red dashed line represents the results after applying a moving average filter to the raw sensor data, with the window size dynamically calculated.

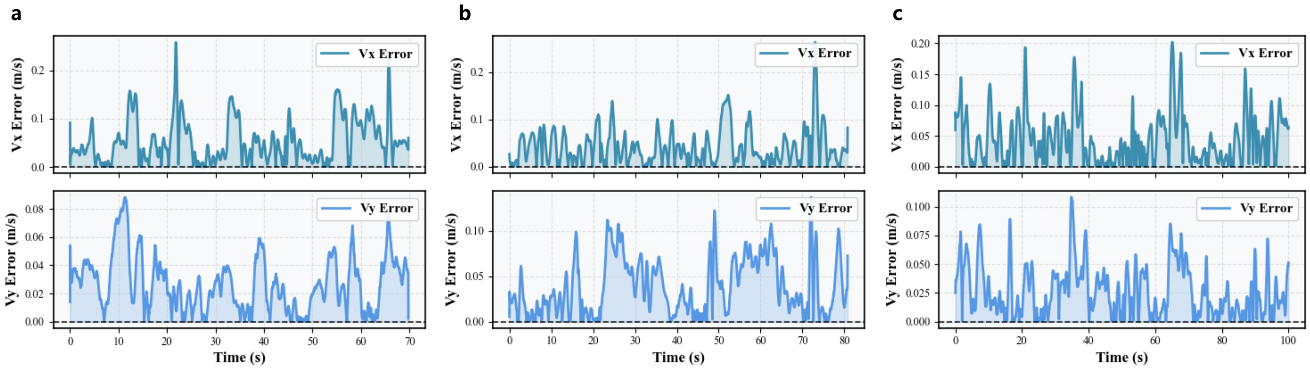

Supplementary Figure 8. Sequence diagram of errors for velocity estimation experiment . (a) Linear motion along the x-axis of the world coordinate system. (b) Linear motion along the y-axis of the world coordinate system. (c) Linear motion in any direction.

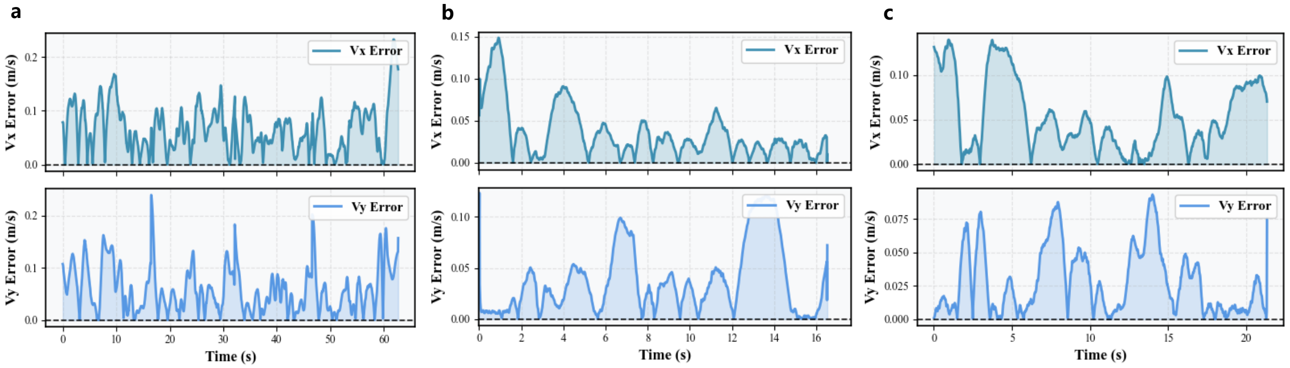

Supplementary Figure 9. Sequence diagram of errors for sensor fusion experiment. (a) Circular motion. (b) Irregular motion 1. (c) Irregular motion 2.

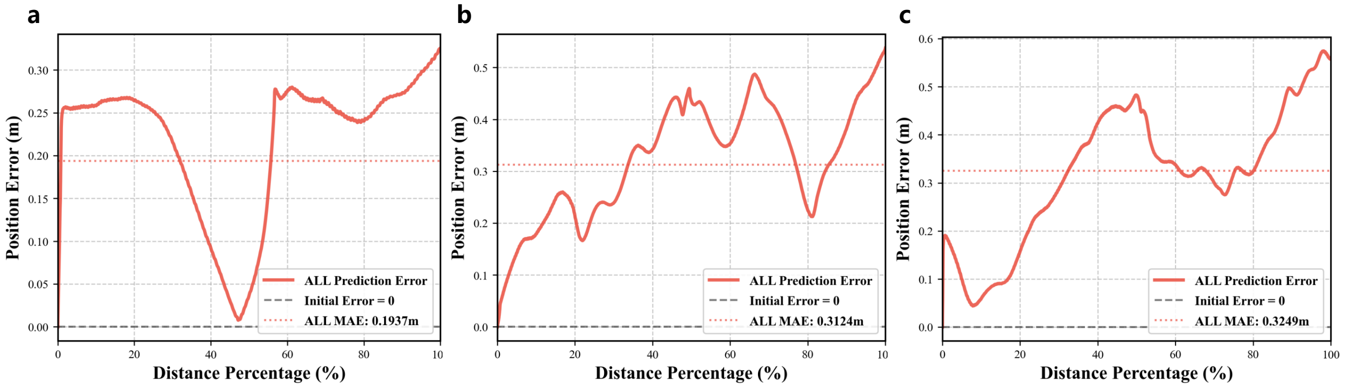

Supplementary Figure 10. Tracking errors for sensor fusion experiment. (a) Circular motion. (b) Irregular motion 1. (c) Irregular motion 2.

Supplementary Videos

The supplementary materials include demonstration videos corresponding to the paper ”Flexible Artificial Lateral Line Based on Luminous Flux for Underwater Velocity Vector Estimation”. There are four videos in total: ”S1 Structure, Fabrication, and Principles.mp4”, ”S2 Relationship Between Force and Sensors.mp4”, ”S3 Velocity Estimation.mp4”, ”S4 Trajectory Prediction.mp4”.

1. **S1 Structure, Fabrication, and Principles.mp4**: It introduces the basic structure, manufacturing process, and operating principles of the propoesd ALL sensor.

2. **S2 Relationship Between Force and Sensors.mp4**: It describes the changes in voltage values under different loading conditions, including step loading tests, principle verification tests, and rotational loading tests.

3. **S3 Velocity Estimation.mp4**: It investigates the predicted velocity changes of the ALL sensor under various ROV motion conditions.

4. **S4 Trajectory Prediction.mp4**: It introduces trajectory prediction for the ALL sensor under various ROV motion conditions.

5. **S5 Flow Field Effects.mp4**: It presents the flow field effects for different types of motion, including flow field contour plots and arrow vector diagrams. The motion types examined include linear motion, circular motion, and irregular motion. The flow field displays information on the relative flow velocity between the ROV and the water current.

Supplementary References

[1] Schlichting H and Gersten K. Fundamentals of boundary–layer theory. *Boundary-layer theory*, 29–49, (Springer2016).

[2] Zhang T, Wang R, Cao Q *et al.* Flowsight: Vision-based artificial lateral line sensor for water flow perception. *IEEE Transactions on Robotics* 2025; **41**: 3260–3277.

[3] Zhang Z, Zhou C, Cheng L *et al.* Real-time velocity vector resolving of artificial lateral line array with fishlike motion noise suppression. *IEEE Transactions on Robotics* 2023; **39**: 4350–4365.

[4] Zhang T, Wang R, Cao Q *et al.* Flowsight: Vision-based artificial lateral line sensor for water flow perception. *IEEE Transactions on Robotics* 2025; **41**: 3260–3277.

[5] He J, Zhou Y, Zhang C *et al.* Online velocity estimation of a robotic fish using artificial lateral line system with velocity-decoupling sensing ability. *IEEE Robotics and Automation Letters* 2025; **10**: 10418–10425.

[6] Tian T, Ma Y, Yu Z *et al.* Bionic lateral-line photonic sensors for high-sensitivity vectorial flow detection and state recognition in underwater environments. *IEEE Transactions on Instrumentation and Measurement* 2025; **74**: 1–10.

[7] Zheng X, Wang W, Xiong M *et al.* Online state estimation of a fin-actuated underwater robot using artificial lateral line system. *IEEE Transactions on robotics* 2020; **36**: 472–487.
